# Single-cell RNA sequencing reveals cell heterogeneity and transcriptome profile of breast cancer lymph node metastasis

**DOI:** 10.1038/s41389-021-00355-6

**Published:** 2021-10-05

**Authors:** Kun Xu, Runtian Wang, Hui Xie, Longfei Hu, Cong Wang, Jiali Xu, Chengjun Zhu, Yiqiu Liu, Fangyan Gao, Xintong Li, Cenzhu Wang, Jinyi Huang, Wenbin Zhou, Guohua Zhou, Yongqian Shu, Xiaoxiang Guan

**Affiliations:** 1 Department of Oncology, The First Affiliated Hospital of Nanjing Medical University, Nanjing, China; 2 Department of Breast Surgery, The First Affiliated Hospital of Nanjing Medical University, Nanjing, China; 3 Singleron Biotechnologies, Yaogu Avenue 11, Nanjing, Jiangsu China; 4 Department of Pathology, The First Affiliated Hospital of Nanjing Medical University, Nanjing, China; 5 Department of Pharmacology, Jinling Hospital, Medical School of Nanjing University, Nanjing, China; 6 Jiangsu Key Lab of Cancer Biomarkers, Prevention and Treatment, Collaborative Innovation Center for Personalized Cancer Medicine, Nanjing Medical University, Nanjing, China

**Keywords:** Tumour heterogeneity, Breast cancer

## Abstract

Molecular mechanisms underlying breast cancer lymph node metastasis remain unclear. Using single-cell sequencing, we investigated the transcriptome profile of 96,796 single cells from 15 paired samples of primary tumors and axillary lymph nodes. We identified nine cancer cell subclusters including CD44 + / ALDH2 + /ALDH6A1 + breast cancer stem cells (BCSCs), which had a copy-number variants profile similar to that of normal breast tissue. Importantly, BCSCs existed only in primary tumors and evolved into metastatic clusters infiltrating into lymph nodes. Furthermore, transcriptome data suggested that NECTIN2-TIGIT-mediated interactions between metastatic breast cancer cells and tumor microenvironment (TME) cells, which promoted immune escape and lymph node metastasis. This study is the first to delineate the transcriptome profile of breast cancer lymph node metastasis using single-cell RNA sequencing. Our findings offer novel insights into the mechanisms underlying breast cancer metastasis and have implications in developing novel therapies to inhibit the initiation of breast cancer metastasis.

## Introduction

Breast cancer is the most common malignancy among women and a frequent cause of cancer-related deaths resulting from metastasis [1]. Axillary lymph nodes are also the most common site for cancer cell migration [2]. Despite extensive research, the detailed mechanisms underlying breast cancer metastasis remain largely unclear. Single-cell RNA sequencing (scRNA-seq) is a technique that can detect the transcriptome profile of isolated cells and has been used to analyze RNA profiles at the single-cell level and to identify cells of a small unique population.

Intratumor heterogeneity is significant in breast cancer metastasis [3]. Cellular composition, gene expression, phenotype modification, and cell-to-cell interactions constitute the microenvironment in which cancer develops and progresses. Cancer stem cells have been regarded as a possible explanation for intratumor heterogeneity and biomarkers that can be used to identify this cluster have been investigated [4–6]. In the present study, we isolated the CD44 + /ALDH2 + /ALDH6A1 + breast cancer stem cells (BCSCs) and proved the pluripotency according to trajectory analysis and RNA-velocity. We also performed genome analysis based on laser-captured microdissected single cells and revealed mutational sites associated with breast cancer lymph node metastasis, including gain in chr8q, chr11q, 12q, and 20q but loss in chr3p, 9p, 11q, 12p, 13q, and 18q [7]. To further investigate metastasis-related mechanisms on a larger scale, we analyzed the copy-number variant (CNV) profile of cancer cell clusters. Based on the inferCNV algorithm, we have testified that BCSCs are originated from the normal breast tissue and exist mostly in primary breast tumors [8]. BCSCs are capable of evolving into more metastatic cancer cell clusters that later infiltrate into lymph nodes.

The previous scRNA-seq has reported that metastatic breast cancer cells highly express genes associated with epithelial-mesenchymal transition (EMT), oxidative stress, proteasome, and biomarkers of cancer stem cells [2, 5, 9, 10]. In a patient-derived xenograft (PDX) model of breast cancer, mitochondrial oxidative phosphorylation was found to be upregulated in micrometastases, while “glycolytic enzymes” were upregulated in primary tumors [2]. Across multiple studies conducted over the years, no single best delineation has been identified to understand the transcriptome diversity of metastatic breast cancer at a single-cell level, nor were comparisons made between primary tumors and lymph node metastases. In the present study, we investigated the cellular composition and transcriptome profiles of five primary tumors and ten paired axillary lymph nodes, demonstrating the heterogeneity of breast cancer, and identified the top differentiated expressed genes (DEGs) associated with breast cancer lymph node metastasis.

The tumor microenvironment (TME) is a complex ecosystem composed of distinct cell populations including immune cells and stromal cells around tumor tissue [11–13]. Cell–cell interactions inside the microenvironment are crucial to understanding the mechanisms underlying tumorigenesis, cancer metastasis, and drug response of cancer cells. Interactions among cell-surface proteins, secreted proteins, and the respective ligand-receptor (L-R) are vital components of the intercellular cross-talk network [14]. We identified cell-to-cell communications that BCSC and metastatic cancer cells take part in and demonstrated the corresponding contributions to lymph node metastasis. For BCSC-immune cell cross-talk, LGALS1-PTPRC, NECTIN2-CD96, and NECTIN2/4-TIGIT are found to hamper immunity against breast cancer; by expressing TIMP-1, BCSC promotes epithelial-mesenchymal transition (EMT) by interacting with CD63 in epithelial cells [15–19]. For lymph nodes with cancer cells, LGALS1-CD69, MIF-CD74, and RPS19-C5AR1 are contributing factors in establishing a pro-tumoral microenvironment [15, 17, 20–22].

To sum up, by delineating the shared and distinct features of cancer cells found in primary tumors and lymph nodes, and by delineating the characteristics of BCSCs, the present study will serve as a useful resource for future studies on tumor heterogeneity and pave the way for individualized treatment for patients with metastatic breast cancers.

## Results

### Single-cell transcriptome analysis of primary breast cancer and lymph nodes

**Fig. 1:**
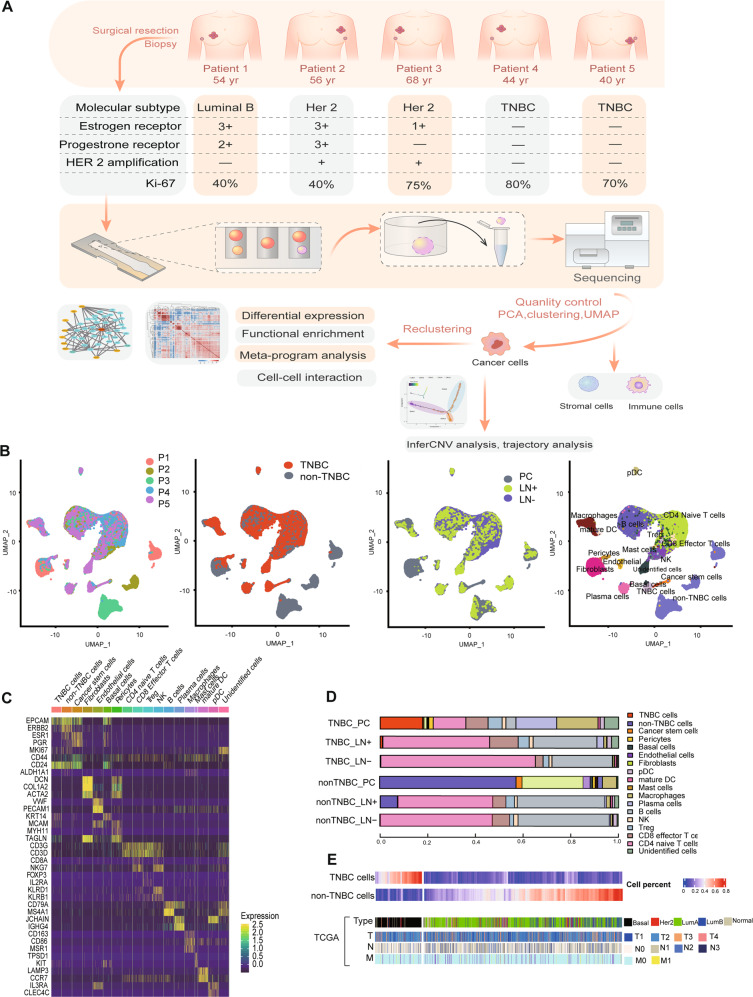
Overview of the transcriptome profile and cell-type classification. **A** Schematic of experimental design and clinicopathological information of samples. **B** UMAP plots of 96,796 single cells from five primary tumors and ten paired axillary lymph nodes, colored according to cell types, patients, molecular subtypes, and sample origins. **C** Heatmap showing expression levels of known cell-type-specific markers. Each column represents a cell. Colors represent cell types as in. **D** Cellular composition of samples according to molecular subtypes and tumor origin. **E** Deconvolution analysis revealing the similarity of samples with TCGA database. Each column represents a TCGA case. Cases with high percentage of cells with our signatures are colored in red. (PC, primary cancer; LN + , lymph nodes with cancer cells; LN-, lymph nodes without cancer cells).

We obtained tissues immediately after therapeutic surgery and axillary lymph nodes dissection from five patients treated in the First Affiliated Hospital of Nanjing Medical University. These tissue samples represent paired primary tumors and lymph nodes from the patients’ axilla of the same side (Fig. 1A). To obtain comprehensive transcriptome landscapes of breast cancer, we performed single-cell RNA sequencing (scRNA-seq) of fifteen samples. After quality control, batch effects removal, and principal component analysis, a total of 27,028 single cells from primary cancer tissue and 69,768 single cells from axillary lymph nodes were visualized using uniform manifold approximation and projection (UMAP, Fig. 1B). We classified all qualified cells into eighteen cell types (Fig. 1B, C). A CD44 + / ALDH2 + /ALDH6A1 + cluster was defined as breast cancer stem cells (BCSCs) [23]. The internal composition of samples was analyzed (Fig. 1D). Deconvolution analysis was applied to analyze the cellular composition of samples in TCGA database, showing the proportion of TNBC and non-TNBC cells in those samples (Fig. 1E). This scRNA-seq data featured the characteristics of TNBC cells that were later applied to define the molecular subtypes TCGA samples, and the results were consistent with the clinicopathological information in the public database. Figure 1E demonstrated the representativeness of our specimens.

### Clonality analysis of breast cancer cells

**Fig. 2:**
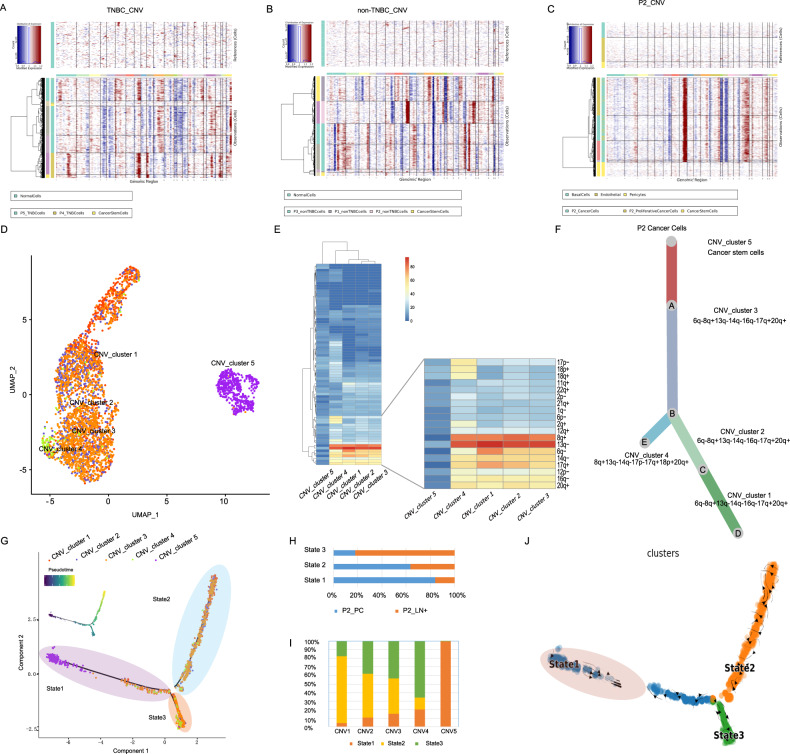
Identification of breast cancer stem cells and evolutionary course of breast cancer. **A**, **B** InferCNV profiles of TNBC (**A**) and non-TNBC (**B**) breast cancer samples. Breast cancer stem cells were characterized with high similarity to normal tissue and presented with few mutations compared with other breast cancer cell clusters. **C** InferCNV profiles of cancer cells originated from Patient 2. **D** UMAP plots of cancer cells originated from Patient 2, colored according to clusters categorized by CNV characteristics. **E** Mutation profiles of CNV_clusters. **F** Evolutionary tree showing the developmental course of five CNV_clusters. CNV_cluster 5 was among BCSC identified in previous research. **G** Pseudotime trajectory demonstrating the transcriptome lineage of five CNV_clusters. Colors indicate pseudotime progression. Cell states are indicated with colored shadows. **H**, **I** Bar plots showing the correlation between evolutionary state and sample origin. **J** RNA-velocity demonstrating the differentiation direction of breast cancer cells.

To probe the genomic profile of breast cancer, we applied inferCNV algorithm to analyze the copy-number variants (CNVs) of a single cell. We found that BCSCs were less mutated compared with other cancer cell subclusters (Fig. 2A, B). Based on the cell counts of BCSCs, Patient 2 was selected for further research. Five CNV_clusters were categorized and CNV_cluster 5 was consistent with BCSC (Fig. 2C and Fig. S1), and the distinct CNV characterization of BCSC was later confirmed by UMAP visualization, in which BCSCs were set apart from the other malignant cells (Fig. 2D). Heatmap demonstrated the mutation profiles of five CNV_clusters according to inferCNV analysis (Fig. 2E). With the increasing investigation in the mechanisms of tumorigenesis, cancer stem cells have been considered as a group of malignant cells originated from mutated normal tissues, possessing intensive activities in self-renewal and differentiation. Descendants of this pluripotent cluster also brought inter-heterogeneity to tumor tissue [4]. Evolutionary study and trajectory analysis was performed to infer the developmental course of breast cancer based on CNV mutation atlas (Fig. 2F, G). BCSCs were identified at an early stage of the trajectory course and evolve into two cancer cell branches with CNV_cluster1 and CNV_cluster4 at the end (Fig. 2F). Our CNV atlas further proved this conclusion on a transcriptomic level, in that CNV_cluster1 and CNV_cluster4 were detected with the most mutations related to lymph node metastasis. Internal composition revealed the relationship between tumor sites and evolutionary state, metastatic cancer cells were at later stages, while the primary lesion held more cells at an early stage (Fig. 2H, I). RNA-velocity testified the stemness of BCSC and evolution along the trajectory course according to the mRNA maturity (Fig. 2J).

### Transcriptome characteristics of metastatic breast cancer cells

**Fig. 3:**
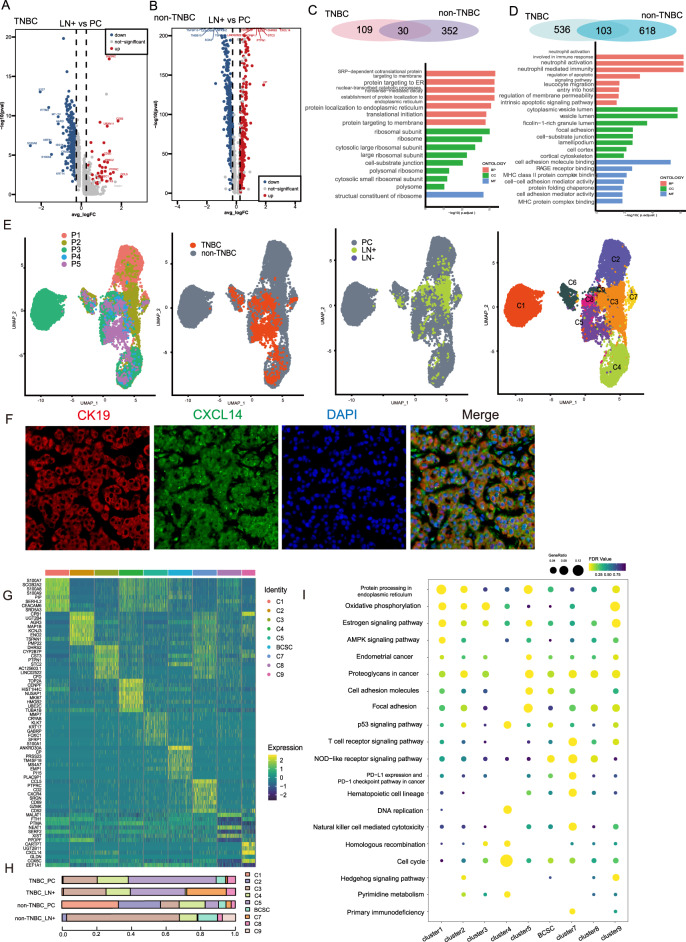
Single-cell transcriptomics reveals distinct cancer cell subpopulations. **A**, **B** Volcano plots of TNBC (**A**) and non-TNBC (**B**) showing differentiated expressed genes of cancer cells derived from lymph node metastasis against cancer cells from primary tumors. **C** Venn plots showing the relationship of upregulated genes in lymph node metastasis of TNBC and non-TNBC. **D** Venn plots showing the relationship of downregulated genes in lymph node metastasis of TNBC and non-TNBC. **E** UMAP plots of cancer cells derived from 15 paired samples of primary tumors and axillary lymph nodes, colored according to patients, molecular subtypes, sample origins, and cancer cell clusters. **F** Immunofluorescence staining showing the differentiated expression of CXCL14 in cancer cells derived from lymph node metastasis. **G** Heatmap showing differentiated expressed genes of nine cancer cell clusters. **H** Cancer cell composition of samples according to molecular subtypes and tumor origins. **I** Gene ontology (GO) enrichment of nine cancer cell clusters.

By zooming in on cancer cells, we dissected DEGs and identified the enriched functions and pathways of primary tumors and metastatic cancer cells, respectively. For metastatic TNBC cells, B2M, CD52, PTMA, and GZMK were among the most significant DEGs, and functional enrichment showed the potential sensitivity for immunotherapy in that immune-related items were highly enriched in metastatic TNBC cells (Fig. 3A). β2-microglobulin, which is encoded by B2M, is an essential component of MHC class I. Previous studies on pancreatic ductal adenocarcinoma, colorectal cancer, and melanoma showed that loss of B2M was an indicator of poor dismal prognosis, including lymph node metastasis, recurrence, and therapy resistance [11, 24, 25]. The high expression of B2M and MHC class I in metastases enabled a better chance for immune recognition and attack. Prothymosin-α, an oncoprotein encoded by PTMA, was found overexpressed specifically in TNBC lymph nodes with cancer cells, which is consistent with the malignancy of cancer infiltration reported by other researchers [26]. For metastatic non-TNBC cells, CXCL14, STC2, CST3, and RAMP3 were overexpressed (Fig. 3B). STC2 expression has been reported to be associated with a longer survival due to less resistance to endocrine therapy and its inhibition of EMT [27, 28]. CST3 expression was reported to be upregulated after DNA damage in TNBC cell lines [29, 30], and showed a positive correlation with trastuzumab resistance in HER-2-positive breast cancers [31]. Previous investigations also revealed that in breast cancer, RAMP3 expression was associated with cancer cell invasion, tumor development, and EMT [32]. Our transcriptome profile demonstrated the heterogeneity across tumor sites and molecular subtypes, and Venn plots showed the inter-tumoral similarities in that thirty genes were found upregulated in metastases of two TNBC and three non-TNBC samples, while 103 genes were found downregulated in both molecular subtypes (Fig. 3C, D). Immunofluorescent staining was conducted to examine the overexpressed genes of non-TNBC cells in lymph nodes (Fig. 3F and Fig. S2). The differentiated transcriptome profiles across tumor sites and molecular subtypes revealed the inter- and intra- tumoral heterogeneity of breast cancer, while some findings echoed research in other solid tumors that indicated the aggressive behaviors of breast cancer cells derived from lymph node metastases.

After the removal of batch effects, UMAP visualized all qualified cancer cells according to breast cancer subtypes, patients, sample origins, and cell clusters (Fig. 3E). Cancer cells from all fifteen samples were filtered and categorized into nine clusters, among which C6 is characterized with BCSC biomarkers including CD44 and ALDH (Fig. 3G). We found that C7 was mostly detected in TNBC lymph node metastases while C3 accounted for the majority of cancer cells in non-TNBC lymph node metastases (Fig. 3H). Referring to the transcriptome profile of C7, CCL5, PTPRC, CD2, CXCR4, and SRGN were among the top DEGs. CCL5 has been known as a contributing factor in TNBC cell proliferation and invasion, as well as in transforming macrophages into the pro-tumoral subtype [33, 34]. Driving invasive motility and metastasis via the TGF pathway, SRGN gene expression was reported to be higher in TNBC cells and tissues than non-TNBC cells and tissues [9]. Moreover, transcriptome analysis on TNBC and non-TNBC revealed an increase in SRGN expression of TNBC lymph node metastases compared with primary tumors. While CXCR4, together with its ligand CXCL12, has been investigated in the mechanisms of breast cancer progression through angiogenesis, EMT, and immunosuppression [35]. Enrichment analysis was conducted to better understand the biological functions of cancer cell clusters, demonstrating the bioactivity in synthesis, metabolism, cell cycle, immune response, and signaling transduction (Fig. 3I). To our knowledge, this is the first study to reveal the distinctions of metastatic breast cancer from a transcriptome standpoint based on integrated single-cell RNA sequencing.

### Identifying malignant programs of breast cancer

**Fig. 4:**
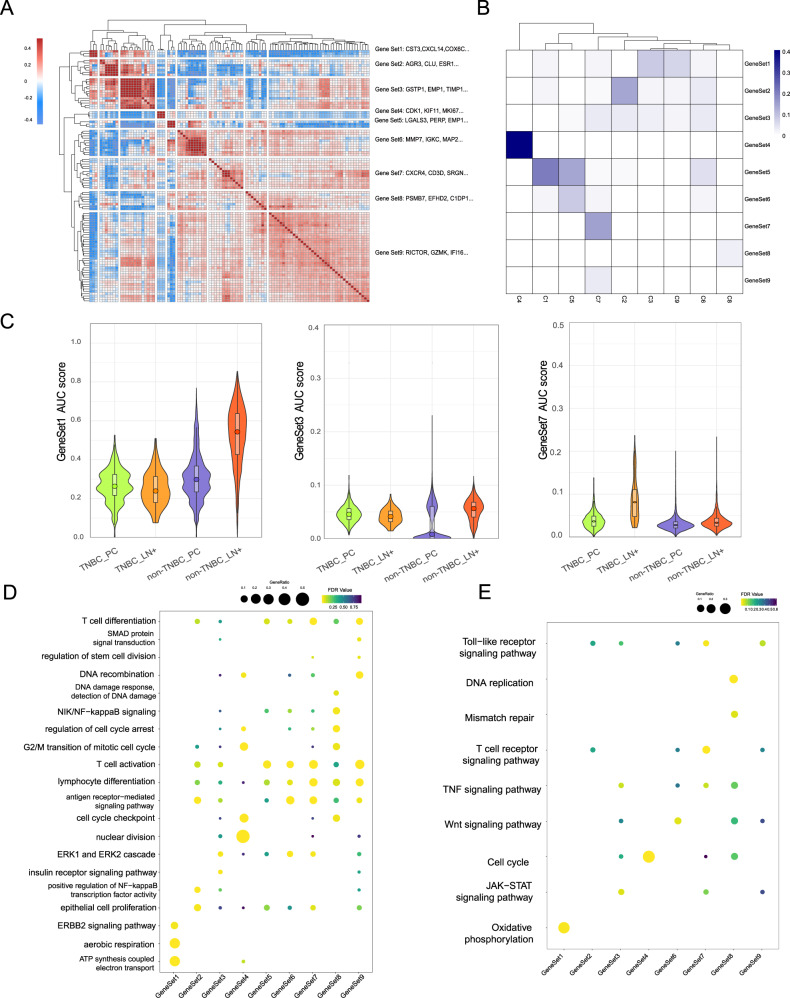
Integrated analysis based on malignant cell identity. **A** Heatmap showing correlation of all programs derived from cNMF analysis of individual patients with breast cancer. Nine highly correlated meta-programs are highlighted. **B** Jaccard similarities of nine meta-programs (y-axis) with the signatures of nine cancer cell types (*x*-axis). **C** Violin plots showing the score of different sample origins in meta-program 1, 3, and 7. **D,** **E** GO (**D**) and KEGG (**E**) analysis of cancer cell clusters.

We applied consensus non-negative matrix factorization (cNMF) to categorize the full transcriptome spectrum of tumoral heterogeneity into nine meta-programs and scored each tumor cell cluster by signature genes of these meta-programs (Fig. 4A–C). Each meta-program corresponded to a gene set with internal similarity or relativity, including energy metabolism (meta-program 1; CST3, CXCL14, COX6C…), cancer-related signaling pathways (meta-program 3; GSTP1, EMP1, TIMP-1…), immune activation and response (meta-program 5; LGALS3, PERP, and EMP1), lymphocyte activation (meta-program 7; CXCR4, CD3D, SRGN…), T-cell activation and DNA repair (meta-program 9; RICTOR, GZMK, IFI16…). Meta-programs provided a better understanding of the heterogeneity and similarity across molecular subtypes and tumor sites of breast cancer.

Composing the majority of lymph nodes with metastatic non-TNBC cells, C3 scored high in meta-program 1. According to functional enrichment (Fig. 4D, E), substance metabolism and ERBB2 signaling pathways were enriched in this meta-program. Unlike metastatic non-TNBC cells, this meta-program analysis demonstrated that metastatic TNBC cells are active in activating immune recognition in the lymph nodes (Fig. 4B, D, and E). The top functions enriched in meta-program 7 included lymphocyte differentiation and activation, and lymphocyte-mediated immunity. Top functions enriched in meta-program 9 included the T-cell receptor signaling pathway, PD-L1 expression and PD-1 checkpoint pathway in cancer, and PI3K-Akt signaling pathway. It has been shown that TNBC is the most immunogenic subtype of breast cancer and immunotherapy including immune checkpoint inhibitors were recommended by the FDA [23]. Our meta-program analysis further showed increased immunogenicity of metastatic TNBC cells, which agrees with that advanced TNBC patients received a more effective response from immunotherapy [36].

### Analyzing the cell-to-cell communications between cancer stem cells and immune cells

**Fig. 5:**
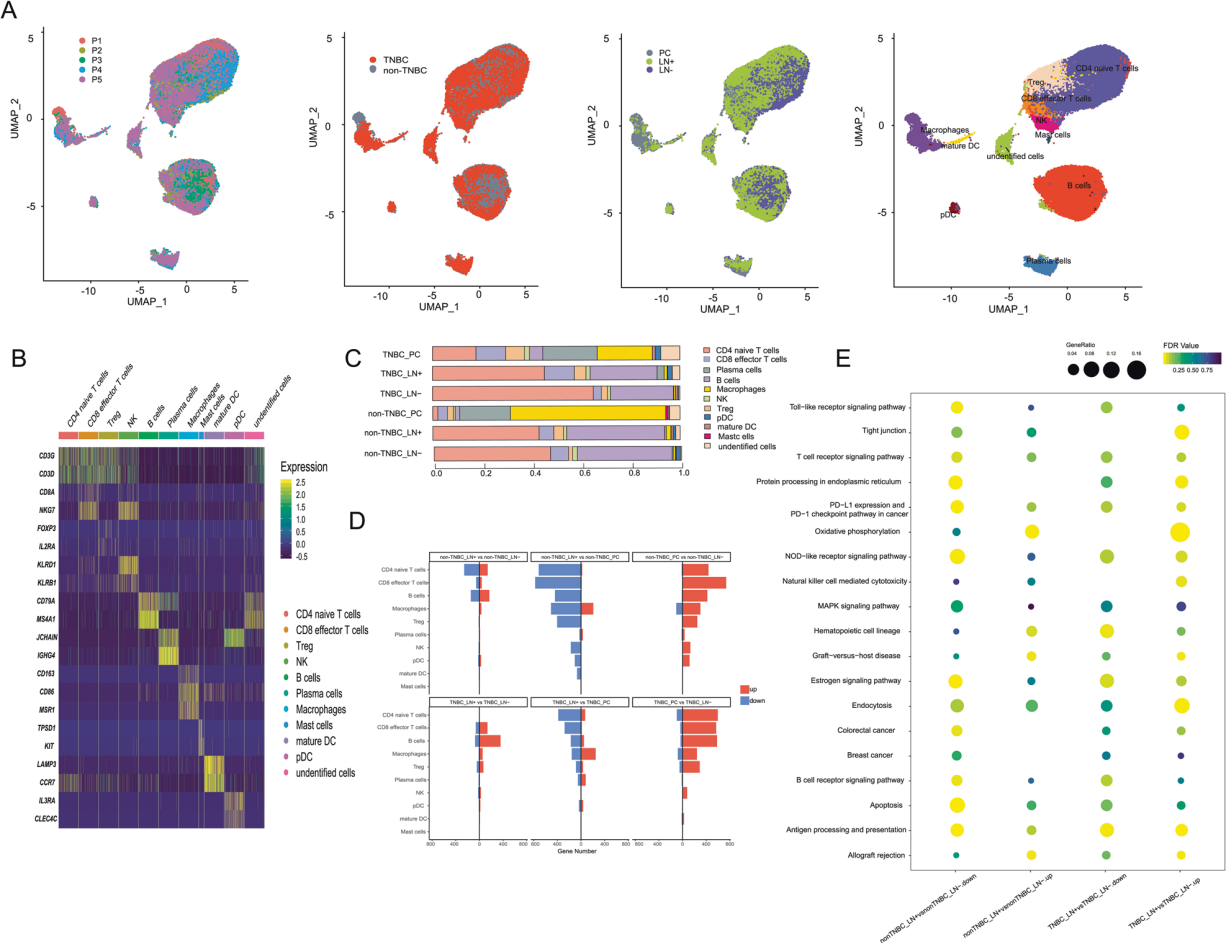
Transcriptome characterization of immune cells surrounding breast cancer cells. **A** UMAP plots of all immune cells from five primary tumors and ten paired axillary lymph nodes, colored according to cell types, patients, molecular subtypes, and sample origins. **B** Heatmap showing expression levels of known cell-type-specific markers. Colors represent cell types as in. **C** Immune cell composition of samples according to molecular subtypes and tumor origin. **D** Bar plots showing comparisons of sample origins in terms of gene numbers. **E** Functional enrichment of transcriptome differentiation across sample origins. (PC, primary tumor; LN + , lymph nodes with cancer cells; LN-, lymph nodes without cancer cells).

**Fig. 6:**
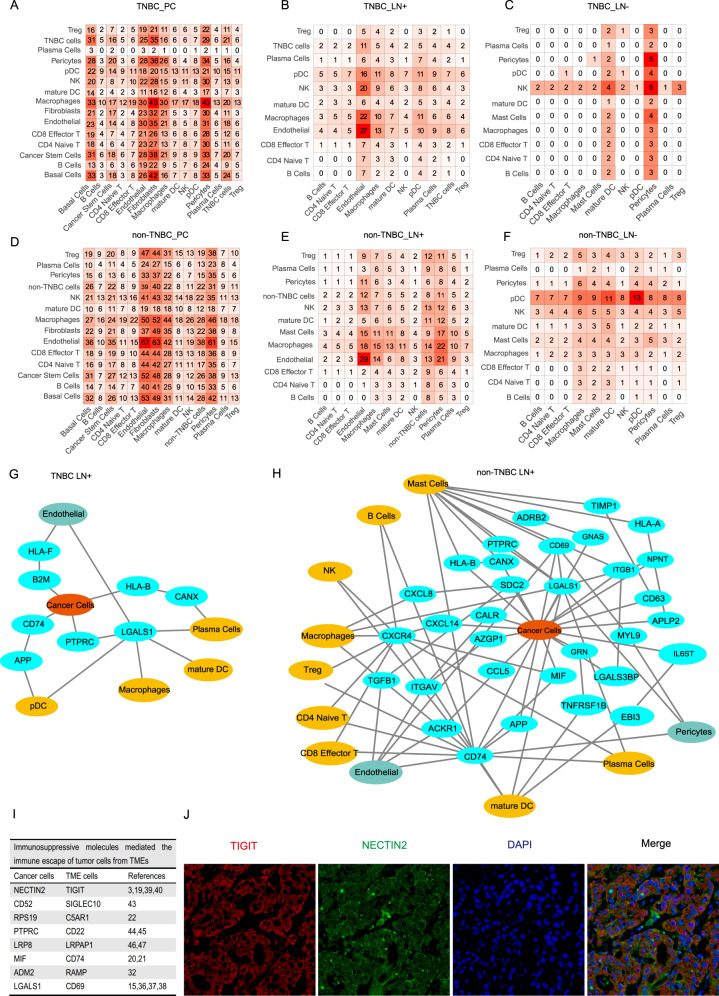
Cell-to-cell communications between cancer cells and immune cells. **A**–**F** Heatmaps showing numbers of cell–cell interactions across cell types from variant sample origins. **G,** **H** NATMI networks showing ligand-receptor pairs among cells in lymph nodes with TNBC (**G**) and non-TNBC (**H**) cells. **I** Table showing impressive molecules and corresponding ligand-receptor pairs associated with breast cancer metastasis and the respective references. **J** Immunofluorescence staining of NECTIN2-TIGIT pair in lymph nodes with cancer cells.

TME is a complex ecosystem composed of distinct cell populations including immune cells and stromal cells around tumor tissue [11–13]. To demonstrate transcriptome characterization of immune cells in the TME, we used UMAP to visualize all the immune cells derived from five primary lesions and ten paired axillary lymph nodes (Fig. 5A, B). TME presented differences in both cellular composition and gene expression across tumor sites (Fig. 5C, D). Biological activities of immune cells are more intense when infiltrating into primary tumor cells compared with those immune cells in lymph nodes, and immune response functioned significantly differently in metastasis across molecular subtypes (Fig. 5D, E). Cell–cell interactions inside the microenvironment are crucial for the mechanisms underlying tumorigenesis, cancer metastasis, and drug response. Cell-surface proteins, secreted proteins, and the respective ligand-receptor interactions are vital components of the intercellular cross-talk network [14]. Based on the Network Analysis Toolkit for Multicellular Interactions (NATMI) analysis, we identified cell-to-cell communications between cell types in different sample origins (Fig. 6A–F).

BCSCs, as a pluripotent cluster, existed in primary lesions of TNBC and non-TNBC, and the NATMI analysis uncovered its function in promoting metastasis through cell-to-cell communication. Encoded by LGALS1, galectin-1 is vital in the tumor-immune escape of breast cancer by binding to the membrane receptor PTPRC on immune cells, and according to previous research [37], PTPRC can cause the apoptosis of activated T cells, promote vascularization of tumor tissue, enhance the function of Treg cells, and attenuate T-cell immune response. Trials on pancreatic cancer revealed that depleted expression of LGALS1 leads to impaired vascularization of the tumor tissue, and restored T-cell immunity [15, 38]. Our single-cell analysis data revealed the expression of LGALS1 in BCSC cluster and the corresponding ligand-receptor pair by which BCSC suppressed the immunity in primary tumors, reinforcing the utility of galectin-1 inhibitor as a potential target therapy against cancer stem cells to control the progression of breast cancer. NECTIN2 and NECTIN4 secreted by BCSC interacted with CD96 and TIGIT on immune cells, causing T-cell inhibition and immune escape at primary breast tumors. TIGIT has been investigated in tumor immunology and antibody-drug conjugate (ADC) targeting NECTIN4 also showed provisional anti-tumor efficacy in preclinical studies [19, 39, 40]. Previous studies revealed a correlation between TIMP-1 expression and poor prognosis in cancer, and the structure-function interaction of TIMP-1 and CD63 has been demonstrated in live cells [41, 42]. BCSCs express TIMP-1 that synthesizes the ligand CD63, further regulates the survival and induces EMT (epithelial-mesenchymal transition) of breast cancer cells [18]. The above-mentioned ligand-receptor pairs were detected among the interactions of BCSCs with immune cells in primary tumors and were found to be associated with immune escape and metastasis of breast cancer cells. Therefore, by combining with inferCNV analysis, we uncovered that BCSCs can not only evolve into metastatic cancer cells but also inhibit immune response and induce metastasis.

### Unraveling the interactions between metastatic cancer cells and immune cells in lymph nodes

The ligand-receptor pairs between metastatic cancer cells and immune cells are crucial components of TME in lymph nodes. CD52-SIGLEC10 and PTPRC-CD22 were involved in both TNBC and non-TNBC lymph node metastases (Fig. 6G, H). CD52 is known as a glycoprotein anchored in mature lymphocytes, the TCGA database showed a higher expression of CD52 in breast cancer cells compared with normal breast tissue, and our samples presented an even higher expression of CD52 in metastatic breast cancer cells of both TNBC and non-TNBC. One study revealed that by binding the inhibitory ligand SIGLEC10, CD52 suppressed T-cell immunity through the damage-associated molecular pattern (DAMP) protein and high mobility group box 1 (HMG1) (Fig. 5B) [43]. Based on this finding, the CD52 antibody alemtuzumab can be a potential therapy specifically targeting metastatic breast cancer cells to further decelerate and even eliminate lymph node metastasis through re-activating T-cell immunity. PTPRC has been identified to bind to the inhibitory receptor CD22 and negatively regulate B-cell immunity [44, 45]. This interaction initiated by PTPRC in breast cancer cells serves as another potential mechanism for immune escape during lymph node metastasis. Besides CD52 and PTPRC that were highly expressed by metastatic cancer cells of both TNBC and non-TNBC. LRP8, an indicator of poorer prognosis was highly expressed in metastatic TNBC cells according to scRNA-seq data [46], and its ligand LRPAP1 was reported to induce T-cell proliferation in leukemia [47]. This interesting finding may explain the significant immunogenicity of TNBC, in that TNBC cells could activate a more intense immune response when migrating to lymph nodes. However, additional mechanisms and biological behaviors remain to be proved in the field of breast cancer. As for metastatic non-TNBC cells, the ADM2 could potentially activate the cAMP pathway by binding RAMP protein on immune cells [32]. Based on DEGs and communications between cancer cells and immune cells in lymph nodes, our findings not only explained the mechanisms of immune escape in breast cancer cells at different sites but also provided targets for future therapies targeting the malignant process of lymph node metastasis (Fig. 6I).

Other interactions, including NECTIN2-TIGIT, LGALS1-CD69, MIF-CD74, and RPS19-C5AR1, were contributing factors in establishing a pro-tumoral microenvironment [15, 17, 20–22]. The MIF-CD74 pair provides a pro-tumoral microenvironment at lymph nodes by impeding M1 polarization of macrophages and chemokine secretion of immune cells as well as by restoring the anti-tumor immunity in the TME and blocking the MIF-CD74 signaling [20, 21]. RPS19 was reported to be overexpressed in breast and ovarian cancer, and its interaction with C5AR1 could induce the secretion of immunosuppressive cytokines and promote the generation of Treg cells [22]. Meantime, in non-TNBC lymph node metastases, LGALS1 expression induced the apoptosis of activated T cells and promoted the angiogenesis of metastatic tumor tissue by interacting with CD69 (Fig. 6H) [16, 38]. By interacting with TIGIT, NECTIN2 was found to be highly expressed in metastatic non-TNBC cells, and by interacting with TIGIT, metastatic cancer cells were found to inhibit T-cell activation and suppress the immune response [19, 39, 48]. We further performed immunofluorescent staining and confirmed this cell–cell interaction between cancer cells and CD8 + effector T cells (Fig. 6J).

We performed scRNA-seq for a comprehensive delineation of the cross-talk between cancer cells and immune cells at primary tumors and metastatic lymph nodes, and the results revealed possible mechanisms of tumor-immune escape underlying breast cancer metastasis and provided therapeutic potentials for the restoration of tumor immunity by targeting specific ligand-receptor pairs between cancer cells and immune cells.

## Discussion

Lymph node status is an important prognostic factor of breast cancer. However, the underlying mechanisms including gene expression profile, intra- and inter- tumoral heterogeneity, and immune escape remain largely unclear, hindering ongoing efforts to target this malignant progress. Previous studies reported gene expression characteristics of breast cancer lymph node metastasis [2, 5, 7]. Across multiple studies conducted over the years, including a tentative exploration of chromatin accessibility during lymph node metastasis derived from one breast cancer patient [24], no single best delineation has been identified to understand the transcriptome diversity of metastatic breast cancer at a single-cell level, nor were comparisons made between primary tumors and lymph node metastasis. The theme of self-renewing cancer cells, epithelial-mesenchymal transition, and tumor-immune escape seem to be recapitulated in research on many malignancies. Despite extensive research, mechanistic relationships between epithelial-mesenchymal transition (EMT), immune inhibition, and BCSC biology have been elusive.

Malignant cells disproportionally arise in tissues and organs that undergo constant growth and self-renewal, and this process led to the hypothesis that the initiation and progression of breast cancer were also driven by BCSCs [49], yet the information about relevant cellular and molecular mechanisms about breast cancer stem cell has been limited. Herein, by using single-cell RNA sequencing to reveal the transcriptome landscape of breast cancer and performing a comprehensive comparison of lymph node metastasis with primary tumors, we isolated BCSC with its unique biomarkers and predicted the evolutionary course among cancer cell clusters. The inferCNV analysis delineated CNV profiles, providing evidence for cancer development course and differentiation of breast cancer cells. We suggested that there might be a possibility for BCSCs to be originated from the normal breast tissue, and alongside the developmental course, cancer cells gained more mutations associated with lymph node metastasis, which is in accordance with research on genomic evolution in previous research [7, 50].

Intratumor heterogeneity has been a key challenge in therapeutic failure and cancer progression [3]. This single-cell transcriptome analysis is analogous to the results from other human malignancies and delineates the transcriptomic heterogeneity of tumors at the single-cell level [12]. Our findings suggest that the expression of genes including PTMA, STC2, CST3, and RAMP3 played contributory roles in lymph node metastasis at the single-cell level [24, 26, 28, 31, 32]. Furthermore, at the gene set level, the meta-program analysis will potentially be used for establishing panels predicting the metastasis and prognosis of breast cancer. Current classification criteria of breast cancer are mainly based on hormone receptor and immunohistochemistry, while our findings open the possibility to predict disease progression with the transcriptome profile, gene set score, and cellular composition of certain cancer cell clusters.

The significance of the tumor microenvironment has been investigated in this study. Cross-talk between immune cells and tumor cells modulated tumor metastasis and therapy response [13]. Our cell-to-cell communication analysis revealed the existence of tumor-immune escape in lymph nodes and unraveled the respective ligand-receptor pairs. We identified that by binding to inhibitory receptors on immune cells, metastatic cancer cells hampered tumor immunity and established a pro-tumoral microenvironment [39]. The NECTIN2-TIGIT interaction between metastatic non-TNBC cells and T cells was confirmed by immunofluorescence staining. Interestingly, though regarded as biomarkers on mature lymphocytes [43], CD52 was found to be highly expressed by metastatic breast cancer cells and contributed to the interaction between breast cancer cells and T cells in lymph nodes by binding to SIGLEC10 [38]. Another study on autoimmune disease revealed the inhibitory effects of PTPRC-CD22 pair [45]. This is the first scRNA-seq analysis to reveal the interactions between metastatic cancer cells and immune cells in breast cancer lymph node metastases.

In summary, we characterized breast cancer stem cells, delineated the evolutionary course of tumorigenesis, identified the transcriptome profiles of metastasis-specific subclusters in breast cancer, and demonstrated the intra- and inter- tumoral heterogeneity in great detail. Our study provides deep insights into future studies of breast cancer lymph metastasis and paves the way for individualized treatment for patients with breast cancer.

## Materials and methods

### Patients and samples

A total of five female breast cancer patients were recruited for this study, including triple-negative breast cancer (TNBC) and non-TNBC (Luminal and HER-2-enriched) cases. All samples were obtained from The First Affiliated Hospital of Nanjing Medical University, Nanjing, China. Three tissue samples of each patient from the primary tumor and two from lymph nodes were collected from each patient after we obtained informed consent. All experimental procedures were approved by the Ethics Committee of The First Affiliated Hospital of Nanjing Medical University and were conducted in compliance with the Helsinki Declaration. Informed consents were obtained from all five patients. Pathologic examination was performed in accordance with the current International Union against Cancer tumor–lymph node metastasis classification. Hematoxylin and eosin (H&E)-stained sections were obtained from primary tumor tissues and paired lymph nodes, then the slices were examined by at least two pathologists (Fig. S3). Histological characteristics, age, metastatic status were evaluated. Primary tumors were classified according to immunohistology staining, and lymph nodes were classified into LN + where cancer cells exist and LN- where no cancer cells were found after quality control according to single-cell sequencing. For P1 (Luminal), P2 (Her-2) and P3 (Her-2), one LN + and one LN- were investigated in this research; for P4 (TNBC), both lymph nodes were LN-; for P5 (TNBC), both lymph nodes were LN + .

### Single-cell RNA sequencing (scRNA-seq)

Five primary tumors and ten paired lymph nodes derived from five patients were used for scRNA-seq. All fifteen fresh tissue samples were collected and immediately stored in the GEXSCOPE Tissue Preservation Solution (Singleron Biotechnologies) at 2–8 °C. Before tissue dissociation, the specimens were washed with Hanks Balanced Salt Solution (HBSS) three times and minced into 1–2 mm pieces. More information about scRNA-seq and analysis was shown in “Supplemental Materials and Methods”. The scRNA-seq data have been deposited in the GEO database under accession code GSE180286.

### Immunofluorescence

Immunofluorescence staining was conducted to prove protein expression and examine the subcellular localization of CXCL14, CK19, NECTIN2, and TIGIT. Tissue biopsies of lymph nodes were deparaffinized and rehydrated, followed by antigen retrieval. After 1-h blocking in 3% bovine serum albumin (BSA)u at 37 °C, tissues were incubated overnight at 4 °C with the following primary antibodies: mouse anti-CK19 antibody (1:200, Abcam Cat# ab7754), rabbit anti-CXCL14 antibody (1:200, Abcam Cat# ab46010), mouse anti-TIGIT antibody (1:200, Thermo Fisher Scientific Cat# 16-9500-82), and rabbit anti-NECTIN2 antibody (1:200, Abcam Cat# ab135246). The secondary antibodies subsequently were added for 1 h at 37 °C followed by counterstaining with DAPI. Tissues were then observed and photographed under the inverted microscope.

## Supplementary information

Supplemental Figure Legends

Supplemental Materials and Methods

FigureS1

FigureS2

FigureS3

## Data Availability

All data needed to evaluate the conclusions in the paper are present in the paper and/or the Supplementary Materials. The scRNA-seq data have been deposited in the GEO database under accession code GSE180286. Additional data related to this paper may be requested from the authors.
