# Supplementary material for: Single-cell RNA sequencing reveals cell heterogeneity and transcriptome profile of breast cancer lymph node metastasis
Source: Oncogenesis. 2021 Oct 5;10(10):66. doi: 10.1038/s41389-021-00355-6 (PMC8492772; doi:10.1038/s41389-021-00355-6)
Supplement: Supplementary file 1 — Supplemental Figure Legends [file 41389_2021_355_MOESM1_ESM.docx]

**Fig. S1 Correspondence between CNV_cluster 1-5 and cancer cell clusters**

Left: transcriptome profiles of cancer cell subclusters;

Right: inferCNV profiles of five CNV_clusters.

**Fig. S2 Immunofluorescence of LN+**

Immunofluorescence of LN+ derived from P2 and P3, showing the expression of CXCL14.

(LN+: Lymph nodes with cancer cells; P2: Patient 2; P3: Patient 3)

**Fig. S3 Pathological staining of fifteen specimens**

Hematoxylin-eosin staining of five primary tumors and ten paired lymph nodes derived from five patients.

(PC: primary tumor; LN 1: Lymph node No.1; LN 2: Lymph node No.2)
