# Supplementary material for: Single-cell RNA sequencing reveals cell heterogeneity and transcriptome profile of breast cancer lymph node metastasis
Source: Oncogenesis. 2021 Oct 5;10(10):66. doi: 10.1038/s41389-021-00355-6 (PMC8492772; doi:10.1038/s41389-021-00355-6)
Supplement: Supplementary file 2 — Supplemental Materials and Methods [file 41389_2021_355_MOESM2_ESM.docx]

**Supplemental Materials and Methods**

**Preparation of single-cell suspensions**

All fifteen fresh tissue samples from five patients were collected and immediately stored in the GEXSCOPE Tissue Preservation Solution (Singleron Biotechnologies) at 2-8°C. Before tissue dissociation, the specimens were washed with Hanks Balanced Salt Solution (HBSS) three times and minced into 1–2 mm pieces. The tissue pieces were digested in 2ml GEXSCOPE Tissue Dissociation Solution (Singleron Biotechnologies) at 37°C for 15min in a 15ml centrifuge tube with continuous agitation. Following digestion, a 40-micron sterile strainer (Corning) was used to separate cells from cell debris and other impurities. The cells were centrifuged at 1000 rpm, 4°C, for 5 minutes and cell pellets were resuspended into 1ml PBS (HyClone). To remove red blood cells, 2 mL GEXSCOPE Red Blood Cell Lysis Buffer (Singleron Biotechnologies) was added to the cell suspension and incubated at 25°C for 10 minutes. The mixture was then centrifuged at 1000 rpm for 5 min and the cell pellet resuspended in PBS. Cells were counted with TC20 automated cell counter (Bio-Rad).

**Single-cell library preparation, and sequencing**

The concentration of single-cell suspension was adjusted to 1×10^5^ cells/mL in PBS. Single-cell suspension was then loaded onto a microfluidic chip and single-cell RNA-seq libraries were constructed according to the manufacturer’s instructions (Singleron GEXSCOPE Single Cell RNAseq Library Kit, Singleron Biotechnologies). The resulting single-cell RNA-seq libraries were sequenced on an Illumina NovaSeq instrument with 150bp paired-end reads.

**Single-cell RNA-seq data processing and quality control**

Raw sequencing data were converted to FASTQ files with Illumina bcl2fastq, version 2.19.1 and aligned to the human genome reference sequence (GRCH38). The CeleScope (https://github.com/singleron-RD/CeleScopeanalysis) pipeline was used to sample demultiplexing, barcode processing, and single-cell gene counting to generate a digital gene-cell matrix from this data. The gene expression matrix was then processed and analyzed by Seurat package [1]. To filter out low-quality cells, we first removed cells for which less than 100 genes were detected or less than 200 UMI, and over 50% of genes derived from the mitochondrial genome. To reduce the influence derived from contamination and doublet in the following analysis, DecontX was used to estimate and remove contamination, and DoubletFinder was used to identify and remove doublet [2]

**Dimensionality reduction, clustering, and visualization**

Seurat v3 was used for dimensionality reduction, clustering, and visualization. For each sample dataset, we used the filtered expression matrix to identify cell subsets. The filtered gene expression matrix was normalized using Seurat’s NormalizeData function, in which the number of UMIs of each gene was divided by the sum of the total UMIs per cell, multiplied by 10,000, and then transformed to log scale (ln (UMI-per-10000+1)). After data normalizing, highly variable genes were identified and used for the following Principal component analysis (PCA). Subsequently, clustering with 20 principal components and resolution 1.0 was performed by graph-based clustering and visualized using t-Distributed Stochastic Neighbor Embedding (t-SNE) or Uniform Manifold Approximation and Projection (UMAP) with Seurat functions RunTSNE and RunUMAP. After cell type identification, immune cells and other cells were extracted and clustered separately for further detailed analysis. The further clustering was performed by Seurat with 20 principal components and a resolution of 0.8.

**Cell types classification**

To identity cell types in sample datasets, we used sets of well-established marker genes for each of those cell types and annotated each cell type based on their average expression and expression ratio. And the type of lymph nodes was confirmed by both H&E staining and scRNA-seq.

**Differential gene expression analysis**

DEGs in a given cell type compared with all other cell types were determined with the FindAllMarkers function from the Seurat package (Wilcoxon rank-sum test, p values adjusted for multiple testing using the Bonferroni correction). For computing DEGs, all genes were probed provided they were expressed in at least 10% of cells in either of the two populations compared and the expression difference on a natural log scale was at least 0.25. DEG were filtered by |fold change| > 0.25 and FDR < 0.05.

**Batch correcting and multiple samples with cancer cell dataset integration**

We applied Harmony integration, which has been shown to reduce batch effects while preserving biological variation for multiple batch integration [3]. RunHarmony returns a Seurat object, updated with the corrected Harmony coordinates. The manifold was subjected to re-clustering use the corrected Harmony embeddings rather than PCs, set reduction = ’harmony’, with parameters of Seurat analysis.

**Inferred CNV Analysis from scRNA-seq**

We identified the malignant cells by inferring large-scale chromosomal copy-number variations (CNVs) in every single cell based on moving averaged expression profiles across chromosomal intervals [4-6]. To run inferCNV, we applied a hidden Markov model (HMM) to predict the CNV level, implemented inferCNV’s i6 HMM model. To compare the CNV change in each cluster, GRCh38 gene information was used to convert each CNV to a p- or q- arm format based on its location. After data conversion, we merged the CNV that belongs to the same arm level. Finally, arms level CNV was annotated as gain or loss. The visualization of the result was performed by heatmap (R package). The display of inferCNV evolutionary plot was drawn by UPhyloplot2.

**Meta program Analysis**

The CNMF was used for transcriptional programs analysis of each sample. We got 20 meta-program for each sample. The top 50 genes with the highest NMF scores were regards as features of the given meta-program. Then calculated the enrichment scores of these 100 gene sets by AUCell [7]. The result was hierarchical clustered based on Euclidean distance after calculation of Pearson correlation coefficient and visualized by heatmap. Finally, we extracted 9 gene sets for the following analysis.

**Jaccard similarity analysis**

The Jaccard similarity coefficient was calculated for comparing the transcriptional similarity between two cell types using their signature genes. We evaluated transcriptional similarity between 9 meta-programs of malignant cells and signatures of 9 cell types/states by calculated Jaccard similarity coefficients using the top 50 marker genes.

**Defining cell scores**

The score of a specific gene set was generated by AUCell, and p values from Wilcoxon rank-sum test were used for estimating the statistical significance between different groups

**Pseudotime trajectory analysis by Monocle and scVelo**

The Monocle2 package (v2.8.0) was used to analyze single-cell trajectories to discover the cell-state transitions. We used the top 100 differentially expressed genes in CNV cluster cells identified by Seurat to sort cells in pseudo-time order. ‘DDRTree’ was applied to reduce dimensions and the visualization functions ‘plot_cell_trajectory’ were used to plot the minimum spanning tree on cells. we get three states of cancer cells. Next, RNA-velocities were predicted using scVelo in the python program.

**Pathway enrichment analysis**

For the differential expressed genes of different cell types, genes with p-value <0.05 and |logFC| > 0.25 were selected for enrichment analysis. The up-regulation genes and down-regulation genes were analyzed separately. The GO and KEGG enrichment analysis was performed by clusterProfiler (3.16.1) [8]. The enrichment analysis of the 9 gene sets generated from CNMF analysis was performed in the same method. The subsequent GSEA analysis of DEGs was performed by GSEApy (https://github.com/zqfang/GSEApy/releases)

**Identification of the correlation of gene expression with the survival of BRCA in TCGA cohorts**

The expression of each gene and the clinical information of each patient with BRCA were obtained from the TCGA cohorts. The coefficient values were obtained from OncoLnc database (http://www.oncolnc.org/download/). The top 20 genes in each cluster of the scRNA-seq data were used for plotting the Cox coefficient values. The average of the Cox values of the 20 genes in each cluster was calculated. In this process, we used MuSiC [9] to perform deconvolution analysis of bulk tumor data (TCGA BRCA) against single cell types.

1 Stuart T *et al*. Comprehensive Integration of Single-Cell Data. *Cell* 2019; 177: 1888-1902 e1821.

2 Dong R *et al*. Single-Cell Characterization of Malignant Phenotypes and Developmental Trajectories of Adrenal Neuroblastoma. *Cancer Cell* 2020; 38: 716-733 e716.

3 Korsunsky I *et al*. Fast, sensitive and accurate integration of single-cell data with Harmony. *Nat Methods* 2019; 16: 1289-1296.

4 Patel AP *et al*. Single-cell RNA-seq highlights intratumoral heterogeneity in primary glioblastoma. *Science* 2014; 344: 1396-401.

5 Tirosh I *et al*. Dissecting the multicellular ecosystem of metastatic melanoma by single-cell RNA-seq. *Science* 2016; 352: 189-196.

6 Venteicher AS *et al*. Decoupling genetics, lineages, and microenvironment in IDH-mutant gliomas by single-cell RNA-seq. *Science* 2017; 355.

7 Aibar S *et al*. SCENIC: single-cell regulatory network inference and clustering. *Nat Methods* 2017; 14: 1083-1086.

8 Yu G, Wang LG, Han Y, He QY. clusterProfiler: an R package for comparing biological themes among gene clusters. *OMICS* 2012; 16: 284-287.

9 Wang X, Park J, Susztak K, Zhang NR, Li M. Bulk tissue cell type deconvolution with multi-subject single-cell expression reference. *Nat Commun* 2019; 10: 380.
