# Supplementary figures and images for: Single-cell RNA sequencing reveals cell heterogeneity and transcriptome profile of breast cancer lymph node metastasis
Source: Oncogenesis. 2021 Oct 5;10(10):66. doi: 10.1038/s41389-021-00355-6 (PMC8492772; doi:10.1038/s41389-021-00355-6)

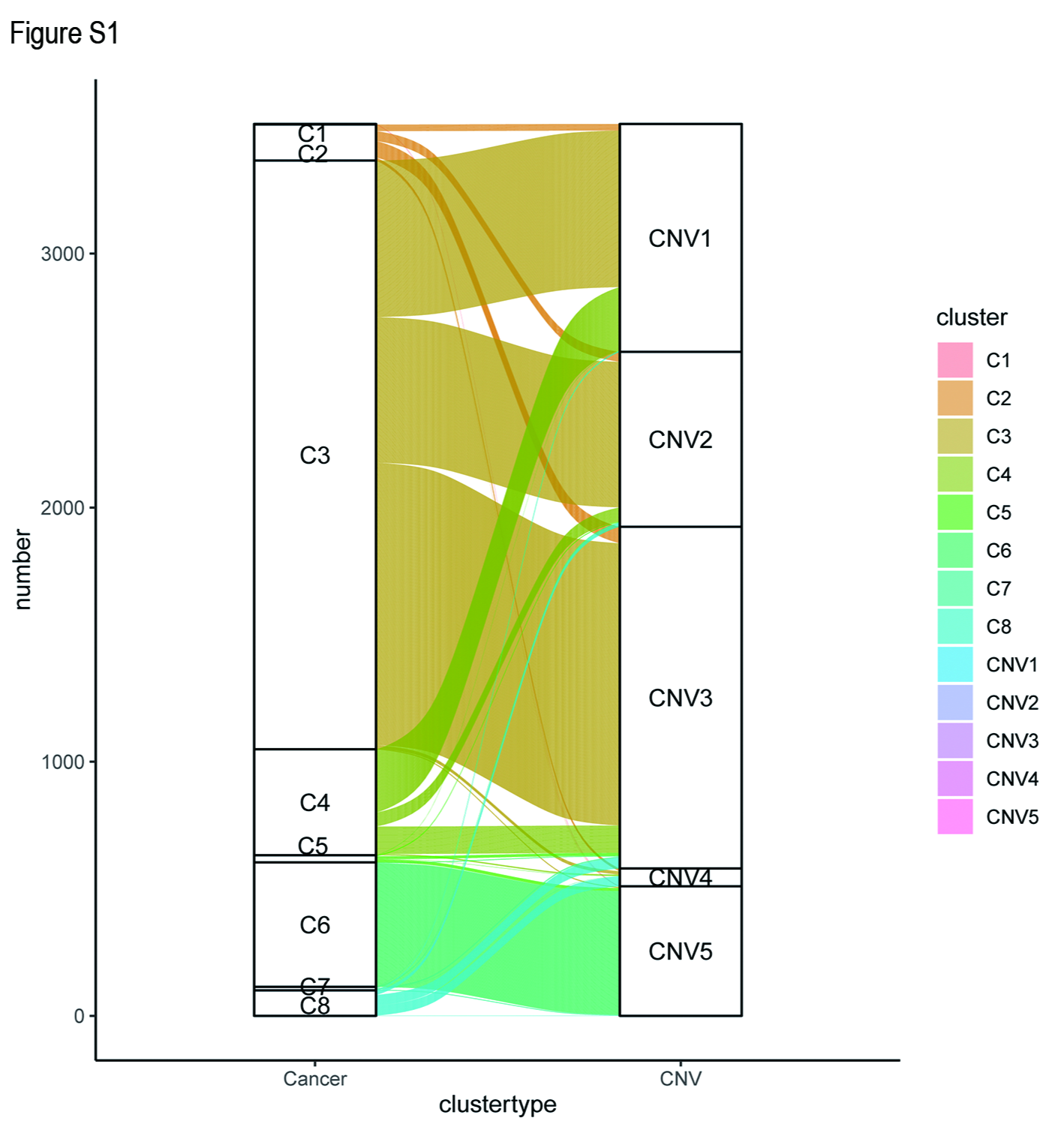

Supplement: Supplementary file 3 — FigureS1 [file 41389_2021_355_MOESM3_ESM.tif]

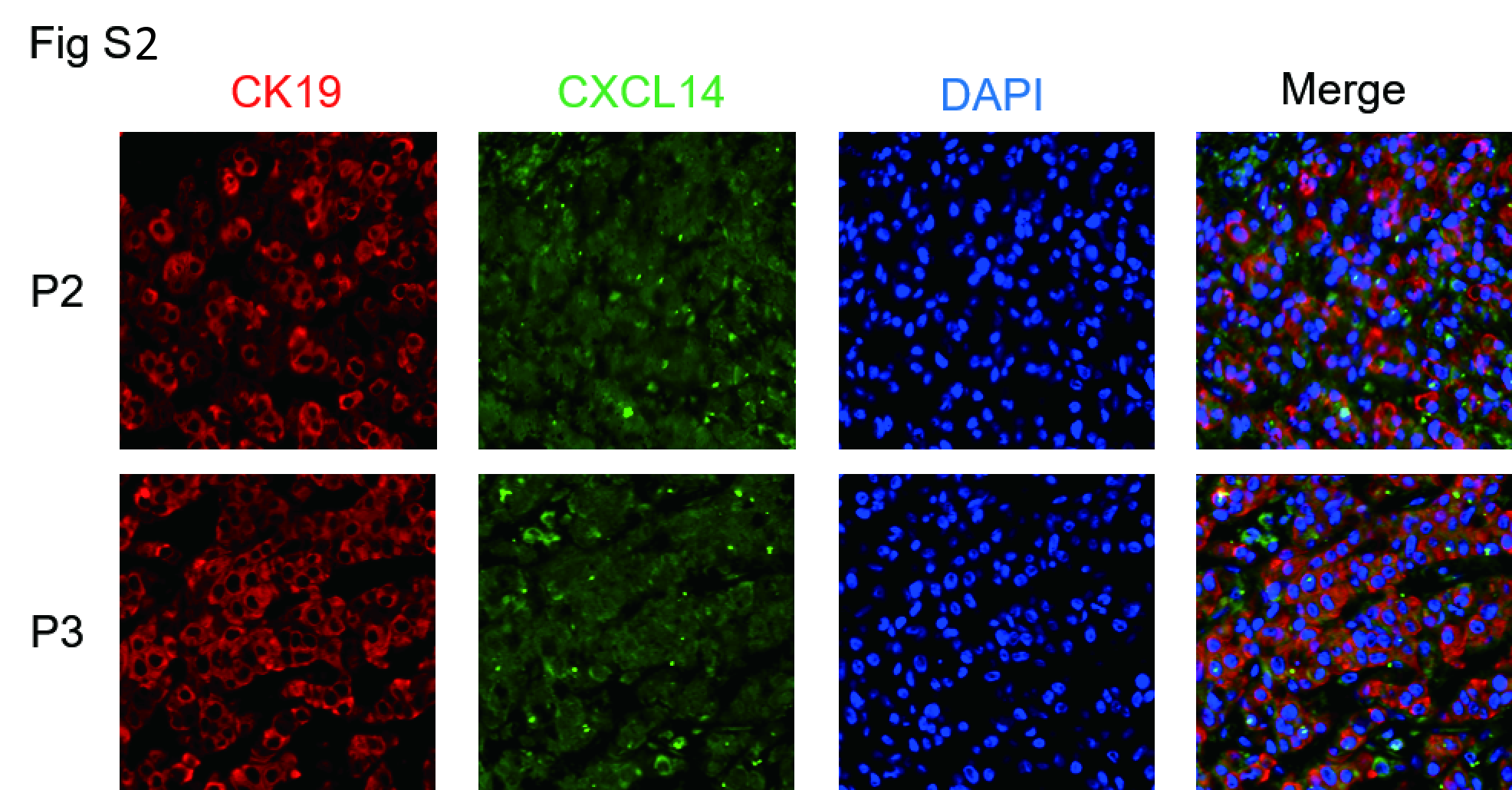

Supplement: Supplementary file 4 — FigureS2 [file 41389_2021_355_MOESM4_ESM.tif]

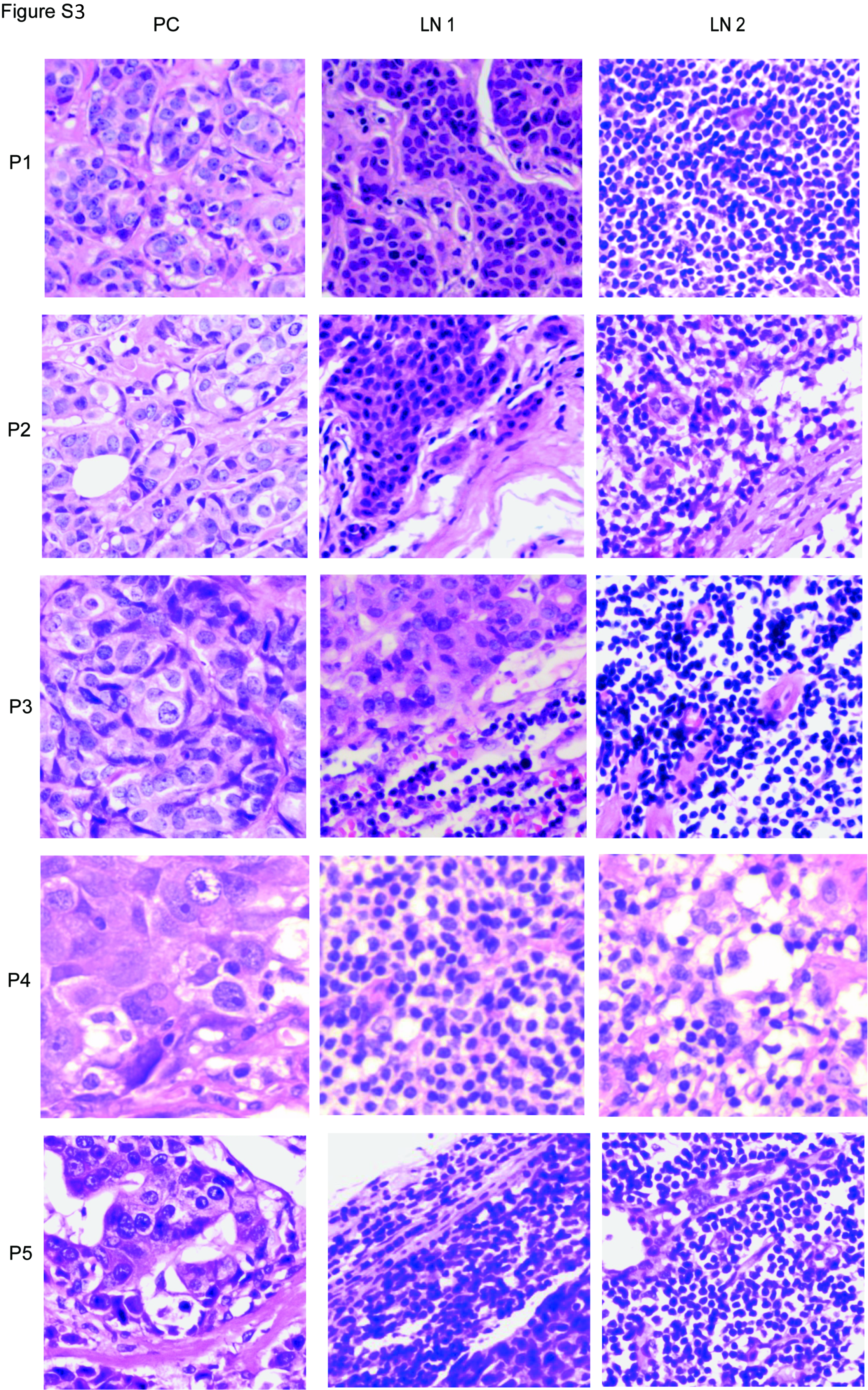

Supplement: Supplementary file 5 — FigureS3 [file 41389_2021_355_MOESM5_ESM.tif]
